# Progress in the Pathogenesis, Diagnosis, and Treatment of TSH-Secreting Pituitary Neuroendocrine Tumor

**DOI:** 10.3389/fendo.2020.580264

**Published:** 2020-11-27

**Authors:** Peiqiong Luo, Lin Zhang, Lidan Yang, Zhenmei An, Huiwen Tan

**Affiliations:** ^1^ Department of Endocrinology and Metabolism, West China Hospital, Sichuan University, Chengdu, China; ^2^ Department of General Practice, West China Hospital, Sichuan University, Chengdu, China; ^3^ Department of Laboratory Medicine, West China Hospital, Sichuan University, Chengdu, China

**Keywords:** thyroid-stimulating hormone-secreting pituitary adenoma, hyperthyroidism, pituitary adenoma, pathogenesis, diagnosis, treatment

## Abstract

TSH-secreting pituitary neuroendocrine tumor (PitNET) is one of the causes of central hyperthyroidism. The incidence of TSH PitNET is far lower than that of other PitNETs. The clinical manifestations of TSH PitNETs mainly include thyrotoxicosis or thyroid goiter, secretion disorders of other anterior pituitary hormones, and mass effect on the pituitary gland and its surrounding tissues. The application of high-sensitivity TSH detection methods contributes to the early diagnosis and timely treatment of TSH PitNETs. Improvements in magnetic resonance imaging (MRI) have advanced the noninvasive visualization of smaller PitNETs. Treatments for TSH PitNETs include surgery, drugs, and radiotherapy. This review focuses on the progress in pathogenesis, diagnosis, and treatment of TSH PitNETs to provide more information for the clinician.

## Epidemiology Overview of TSH PitNET

Pituitary neuroendocrine tumor (PitNET) is the most frequently intracranial tumor, and the occurrence seems to be similar despite geographic or ethnic differences (1). TSH PitNETs are the rarest among the PitNETs, only accounting for 0.5% to 3% of all PitNETs (1). The incidence is mainly sporadic, and a few familial clusters have been reported in patients with multiple endocrine neoplasia type 1 (MEN-1). TSH PitNETs are relatively rare, with less than 500 cases reported at present. According to epidemiological surveys, the incidence is approximately 0.3 ~ 1.0/1 million. Statistics in northern Europe (such as Finland and Sweden) show that the incidence of TSH PitNETs is about 0.26 ~ 0.32/1 million per year (1). Another study from the Swedish National Pituitary Registry found a national prevalence of 2.8 clinically overt cases per million inhabitants and an incidence rate of 0.15 cases per million inhabitants per year (2). There is still a lack of epidemiological data in China (1).

TSH PitNETs are the main cause of central hyperthyroidism. In 1960, Jailer et al. (3) first reported patients with TSH PitNET showing clinical symptoms of hyperthyroidism and typical imaging manifestations such as sella turcica enlargement. The onset age of TSH PitNETs can range from 8 to 85 years old, usually at 40 ~ 50 years old. TSH PitNETs are more common in adults, with more females than males (4). There have also been a few reports of TSH PitNETs in children and adolescents (5). TSH PitNETs are often misdiagnosed as primary hyperthyroidism. Unfortunately, some patients with TSH PitNET were misdiagnosed as Graves’ disease and received unnecessary iodine-131 radiotherapy or even thyroidectomy. What is worse, inappropriate treatment may promote the growth of tumors (6).

Recently, with the increasing sensitivity of TSH detection technology and the widely used magnetic resonance imaging (MRI) and other imaging technologies, the detection and diagnosis rates of TSH PitNETs have significantly increased (7). According to publications of the latest medical guidelines for TSH PitNETs, an increasing number of clinicians are concerned about it (8).

## Pathogenesis, Histopathology, and Molecular Characteristics of TSH PitNETs

TSH-secreting cells account for less than 5% of all pituitary cells, which may explain why TSH PitNETs are so rare (8). Thyrotrophs are widely distributed and are mostly concentrated in the anteromedial part of the pituitary gland, so most invasive TSH PitNETs are located in the middle of the pituitary gland (5). PitNETs were classified according to a combination of pituitary transcription factors (Pit-1, ER - 3, SF-1, Tpit, et al), hormones, and other biomarkers (keratin, Ki67, p27, FGFR4, et al). There are at least three types of TSH PitNETs (9–11).

The first one is thyrotroph tumor. By light microscopy, arranged in cords, tumor cells are chromophobic and polymorphous (usually polyhedral or angular), with large nuclei and prominent nucleoli. By electron microscopy, the tumor cells are elongated and possess elongated cytoplasmic processes, with moderately developed rough endoplasmic reticulum (RER) and Golgi complexes. Small secretory granules are arranged peripherally under the plasmalemmae. Thyrotroph tumors express Pit-1, GATA-2/3, TSHβ, and/or α-subunit (α-SU), and they do not express other hormones (9, 12). Fibrotic characteristics are seen in approximately 40% of the thyrotroph tumors, which is different from other PitNETs and make it slightly firm. The higher the degree of fibrosis, the denser the thyrotroph tumor is, and some may even appear calcified. It is reported that basic fibroblast growth factor (bFGF) may mediate the process of fibrosis, which is a potent mitogenic angiogenic factor and is normally expressed in pituitary folliculostellate cells. At nonmitogenic levels, bFGF regulates TSH, growth hormone (GH), and prolactin (PRL) secretion.

The second one that secret TSH is poorly differentiated Pit-1 lineage tumors, previously known as silent subtype 3 PitNET. By light microscopy, tumor cells are usually chromophobic or eosinophilic, with mild to moderate pleomorphism and occasionally prominent nucleoli. Electron microscopy can show characteristic nuclear spheridia, secretory granules, increased RER amounts, a diffusely expanded and tortuous Golgi apparatus. They express Pit-1, GATA-2/3, estrogen receptor-alpha (ER-a), a-SU, TSHβ, GH, PRL, and/or CAM5.2 (± fibrous bodies) and usually do not have a consistent pattern of hormone expression. They are highly aggressive and invasive (9, 13, 14).

The third one that secret TSH is plurihormonal tumors of Pit-1 lineage, including GH-producing plurihormonal tumors. By light microscopy, tumor cells are usually monomorphous (uniform, polyhedral or elongate) and acidophilic in a diffuse, trabecular, or sinusoidal pattern. Electron microscopy can show tumor cells with numerous mature secretory granules, a predominantly spherical or ovoid nucleus, well-developed RER, and prominent Golgi apparatus (9) . They express Pit-1, ER-a, GATA-2/3, GH, PRL, TSHβ, and/or α-subunit (12).

In TSH PitNETs, cosecretion with GH and PRL is common, which might exist in 16% and 10% of cases, respectively. The cosecretion with luteinizing hormone (LH) or follicle-stimulating hormone (FSH) is rare. No cosecretion with ACTH(Adrenocorticotropic Hormone) has been reported so far (15).

The cells of thyrotroph tumors and TSH plurihormonal tumors of Pit-1 lineage express somatostatin receptor (SSTR), including SSTR1, SSTR2, SSTR3 and SSTR5. Among these, SSTR2, and SSTR5 are the most common subtypes (16). TSH PitNETs are mostly of monoclonal origin, similar to other PitNETs. TSH PitNETs are derived from a single cell that transforms into a tumor cell and then amplifies monoclonally. Mutations in proto-oncogenes (Ras, protein kinase C, G-protein subunits, TRH receptor, gsp) and tumor suppressor genes (p53, Rb, MEN-1) in TSH PitNETs have not been found (14, 17). The loss of heterozygosity (LOH) of MEN1 gene locus (11q13) was found in a few cases, but a concurrent mutation of MEN1 gene was not found (14, 18) . In a report from six Belgian and French centers, where 43 patients with TSH PitNETs were described, 2 patients were diagnosed with clinical MEN-1(multiple endocrine neoplasia type 1), but no MEN-1 mutation was found, which indicates that TSH PitNETs may be the pituitary component in MEN-1 (14, 19) . Germ-line mutations in the aryl hydrocarbon receptor-interacting protein (AIP) gene have been reported in patients with TSH PitNET (20). Zhang et al. (21) found increased expression of the Wnt4 gene in TSH PitNETs, in comparison with normal pituitary glands, which indicated that the Wnt4 gene may be related to the pathogenesis. In recent studies, due to the tumor’s resistance to triiodothyronine inhibition, mutant forms of thyroid hormone receptors(TR) may be potential candidate oncogenes. It has been reported that the absence of TRα1, TRα2, and TRβ1 expression in two patients with TSH PitNET. The aberrant expression of TRβ4 may be partly related to the inappropriate secretion of TSH in TSH PitNETs. The mechanism of the chromosome in the pathogenesis of TSH PitNETs is also unclear. Recurrent gains in chromosomal arms 1q, 4q, 5p, and 19q were reported (18).

In addition, the circadian rhythm of TSH secretion is also an important research direction for understanding the pathogenesis of TSH PitNETs. Roelfsema et al. (22) confirmed that the rhythmicity of TSH secretion was related to the abnormal secretion of TSH. Recent studies have found that the abnormality of negative feedback regulation in the pituitary-thyroid axis is related to defective products after TR mRNA transcription (23). At present, the etiology of TSH PitNETs has not been fully understood and still needs further study (5, 17, 23).

Malignant transformation is very rare. Whether TSH PitNET is malignant is mainly decided by the presence of metastasis. TSH is the major growth factor for thyroid cells, and many experimental studies suggest that it could play a role in carcinogenesis of thyroid tumors. The exact clinical impact of this continuous TSH stimulation on thyroid gland has not been well confirmed. In animal experiments, it has been found that phosphoinositide 3-kinase (PI3K) may be related to its carcinogenesis (24).

## Diagnosis

Most patients with TSH PitNETs have developed enormous tumors by the time they see a doctor, which may have caused damage to the surrounding dural space and the sella turcica bone. Therefore, early diagnosis is particularly important. The diagnosis of TSH PitNETs requires a combination of clinical manifestations and signs, endocrine hormone measurements, functional tests, and imaging findings. When the levels of serum FT4 and FT3 are higher than the normal range, and the serum TSH level is not inhibited, the possibility of the existence of a TSH PitNET should be suspected after excluding interference from the laboratory testing technology. We can refer to the two steps of qualitative diagnosis and localization diagnosis to realize a standardized diagnosis. The qualitative diagnosis relies on family history, clinical symptoms and signs, biochemical tests, and a series of dynamic tests mentioned below. The localization diagnosis relies on imaging tests, including MRI, CT, scintigraphy, and others. It is noted that since the pathogenesis is not clear at present, an etiological diagnosis cannot be achieved yet.

## Clinical Symptoms and Presentation

The majority of TSH PitNETs have an insidious onset and a chronic course. The clinical manifestations of TSH PitNETs mainly include the following three aspects.

### Thyrotoxicosis or Thyroid Goiter

Hyperthyroidism at the clinical level has been reported in 67% of patients (25). For the functional TSH PitNETs, excessive TSH secretion leads to an increase in the synthesis and secretion of thyroid hormone by the thyroid, which leads to clinical manifestations of different degrees of thyrotoxicosis, including heat intolerance, hyperhidrosis, palpitations, emaciation, irritability, sleep disorders, and others. TSH can induce thyroid enlargement to different degrees or even thyroid nodules. It has been reported that the incidence of diffuse or nodular thyroid enlargement in patients with TSH PitNET is approximately 70% (26). The clinical manifestation of thyrotoxicosis in patients with TSH PitNET is mostly mild to moderate and is usually not consistent with the hormone levels. It is rarely accompanied by manifestations of the autoimmune thyroid diseases such as protrusion of the eyes, myxedema, and thyroid toxic symptoms such as atrial fibrillation and heart failure.

In addition to functional TSH PitNETs, there is another type of tumor that is clinically “silent”, with higher invasiveness and recurrence rate. TSH PitNETs are usually found incidentally or due to symptoms caused by mass effect. Immunohistochemistry (IHC) can detect hormone secretion, even when there is no symptom or sign of hormone excess. “Totally silent” tumors show no increased basal and stimulated TSH serum concentration. “Clinically silent” tumors show slightly increased TSH serum concentration. About half of silent TSH PitNETs present with extra-sellar extension, and the most common symptoms are visual impairment and headache. They expressed SSTRs, so somatostatin analog treatment was effective. In silent TSH PitNETs, mild or atypical clinical presentations tend to be overlooked, and endocrinologists should be vigilant (27).

### Increased or Decreased Secretion of Other Hormones From the Anterior Pituitary Hormones

Cosecretion with other pituitary hormones accounts for about 42% of TSH PitNETs. The most common situation is the hypersecretion of GH, which leads to acromegaly or gigantism. The second most common situation is the hypersecretion of PRL, which mostly occurs in women and leads to the galactorrhea-amenorrhea syndrome. Most women with mixed TSH, PRL PitNETs, or simple TSH PitNETs show menstrual disorders. Men may have a decreased libido. Children and adolescents may have delayed sexual development. The symptoms of hyperthyroidism may be masked by acromegaly or galactorrhea-amenorrhea syndrome and can be ignored (28). On the other hand, when TSH PitNETs is too large, it can compress and infiltrate the pituitary gland and its surrounding tissues, leading to the insufficient secretion of other hormones from the anterior pituitary, especially LH and FSH. Partial or total hypopituitarism can be seen in about 25% of patients (15).

Hyperprolactinemia is mostly found in non-thyrotroph TSH PitNETs, including poorly differentiated Pit-1 lineage tumors and plurihormonal tumors of Pit-1 lineage other than thyrotroph tumors. The main reason is that Pit-1 is the transcription factor responsible for regulating PRL production. To note that, other situations, including TRH excess, stalk effect and et al. causing hyperprolactinemia should be excluded. First, increased TRH caused by primary hypothyroidism can promote PRL secretion in lactotroph cells, usually with PRL values <100 µg/L. In contrast, TSH PitNETs often show a low TRH level, which is inhibited by increased TSH. Second, diseases which compress pituitary stalk and portal vessels (Rathke’s cleft cyst, craniopharyngioma, meningioma, lymphocytic hypophysitis, sarcoidosis, empty sella syndrome, et al.) can lead to hyperprolactinemia, usually with PRL level <100 µg/L. Pituitary MRI may also help differential diagnosis. In addition, the level of PRL in TSH PitNETs usually over 100 µg/L; while in the above situations including TRH excess, stalk effect, the counterparts are usually no more than 100 µg/L. Moreover, TSH PitNETs have no obvious response to PRL stimulant (TRH, cimetidine) or inhibitor (levodopa), which can also help differential diagnosis (29).

### Mass Effect on the Pituitary Gland and Its Surrounding Tissues

The mass effect accounts for 29%–38% of onset presentation in patients with TSH PitNET (8). TSH PitNETs, especially an enormous tumor, can lead to the impairment of anterior pituitary function and relative symptoms. Patients may show symptoms including eye distension, vision loss, visual field defect, headache, nausea, vomiting, increased cranial pressure, and even pituitary apoplexy (30).

## Biochemical Characteristics

### Basic Biochemical Tests

Due to the use of ultrasensitive TSH methods since the late 1980s, the diagnosis of TSH PitNETs has evolved substantially and is more frequently found when the tumor size is less than 1.0 cm (8). The characteristics of endocrine hormone in TSH PitNETs are an elevated thyroid hormone level and a normal or elevated TSH level. Hyperthyroidism at the biochemical level is reported in 90% of the patients (25). The diurnal rhythm was observed in TSH PitNETs and a rare case of TSH PitNETs with cyclic fluctuations in serum TSH levels has been reported recently (31). In addition, approximately 30% of the patients also showed an elevated level ofα-SU. Some reports found that α-SU level may be related to TSH PitNETs’ diameter.et, al Moreover,α-SU may also increase in some women with primary hypogonadism (8). Other substances such as sex hormone binding globulin (SHBG), cholesterol, angiotensin-converting enzyme, soluble interleukin-2 receptor, osteocalcin, carboxy-terminal cross-linked telopeptide of type I collagen (ICTP) can also be used as clinical indicators for the evaluation of hyperthyroidism. Elevated ICTP illustrates the hypermetabolic state in the bone in patients. The level of SHBG may be normal in mixed GH/TSH PitNETs, because GH may impose an inhibitory impact on SHBG synthesis and secretion. Moreover, other pituitary hormones such as GH, PRL, FSH, and LH can also be expressed when TSH PitNETs are plurihormonal tumors. In addition, nonfunctioning pituitary tumors(NFPA) occur in 10%–20% of the normal population, which the increased TSH cannot be detected, and thus we cannot exclude TSH PitNETs simply if the result of the biochemical tests is negative (8).

### Dynamic Tests

Thyroid-releasing hormone (TRH) stimulation test can also help diagnosis, which requires a rapid intravenous injection of 400–500 µg TRH, followed by the measurement of the level of TSH. An increase in the TSH level of less than 5 uIU/ml or less than 2 times in comparison with the base value is defined as no reaction. An increase in the TSH level of approximately 5–35 uIU/ml or 3–5 times in comparison with the base value is defined as a normal reaction. An increase in the TSH level of more than 35 uIU/ml or more than 5 times in comparison with the base value is defined as an enhanced reaction (32). Approximately 90% of TSH PitNETs show no reaction to the TRH stimulation test. In addition, about 44% of patients can show an increased α-SU level after TRH stimulation (33). The sensitivity of the TRH stimulation test is poor in patients with a history of thyroidectomy. In China, since the TRH experimental drug cannot be obtained, the test cannot be carried out at present.

The classic T3 inhibition test is also used to diagnose TSH PitNETs. This test is abnormal in nearly 100% of TSH PitNETs (1). This test has good sensitivity in patients with a previous thyroid surgery history. If the patient has been thyroidectomized, the result can be contradictory. T3 inhibition test is the most sensitive and specific test to evaluate whether the tumor has been completely removed. This test can also be used to exclude nonfunctioning pituitary tumors and differentiate TSH PitNETs from secondary pituitary hyperplasia. It should be noted that old age and a history of coronary heart disease are absolute contraindications for a T3 inhibition test. However, due to the complex process, a large amount of time required, and large individual differences, the clinical application of this test is not common.

The somatostatin inhibition test is also recommended for the diagnosis of TSH PitNETs. Most patients with TSH PitNET showed a significant decrease in the level of TSH, FT3, and FT4 after injection of a somatostatin analogue (30). At present, Sandostatin LAR (octreotide acetate microsphere for injection) and Sandostatin (octreotide acetate injection) are the somatostatin analogues most commonly used.

Moreover, some cases with TSH PitNET showed a significant increase in the serum TSH concentration in response to growth hormone-releasing peptide-2 (GHRP-2), which stimulates the release of TSH *via* the GHRP receptor type 1α. However, the mechanism of this test is not entirely clear, and the test is not used widely=.

## Imaging Manifestations

**Figure 1:**
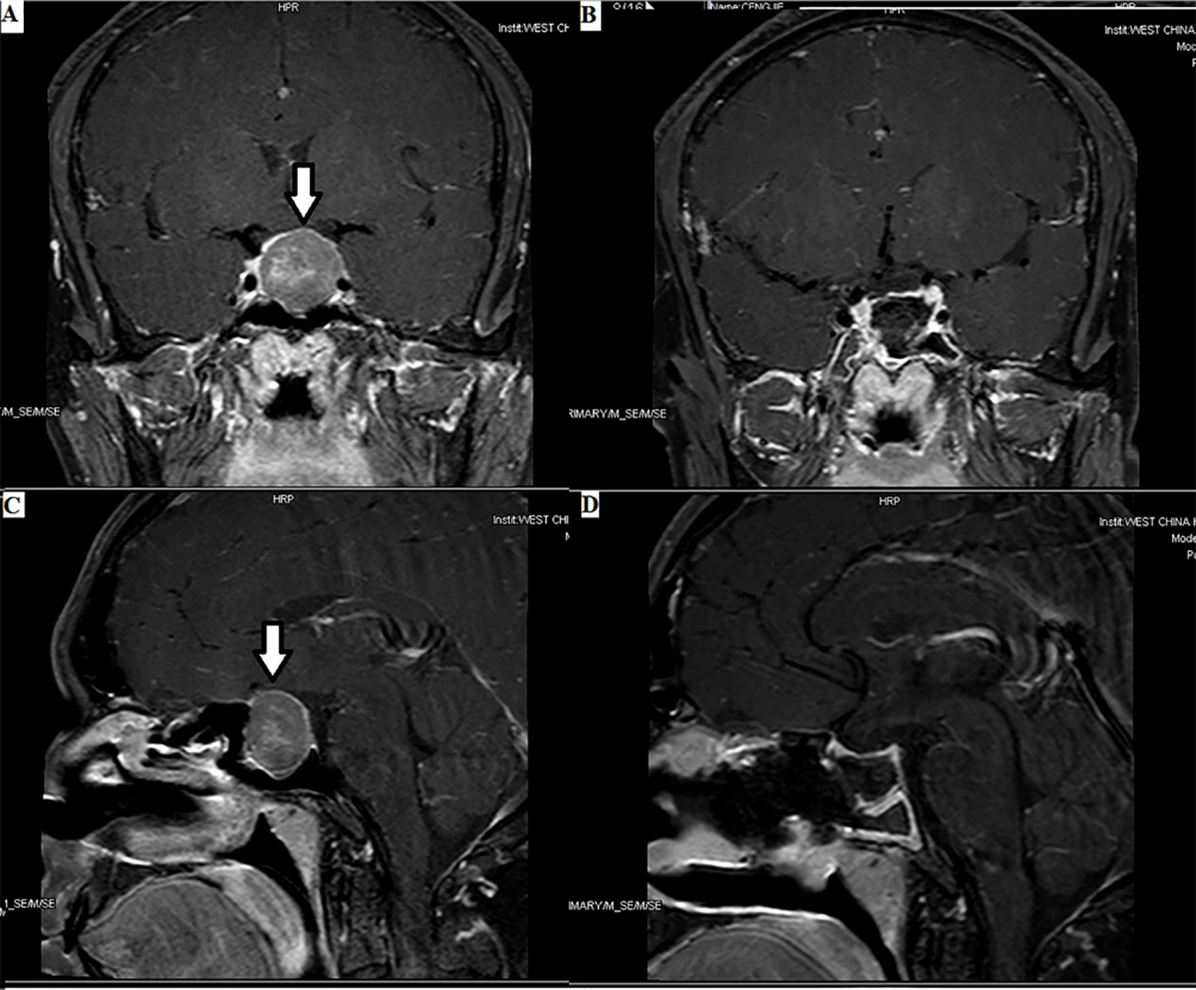
Enormous TSH PitNET: 1. Preoperative magnetic resonance imaging (MRI) **(A, C)**: solid tumor occupying the saddle area, with a size of 1.8×2.4×2.5 cm, as indicated by the arrows. 2. Postoperative MRI **(B, D)**: low signal shadow in the sella region.

**Figure 2:**
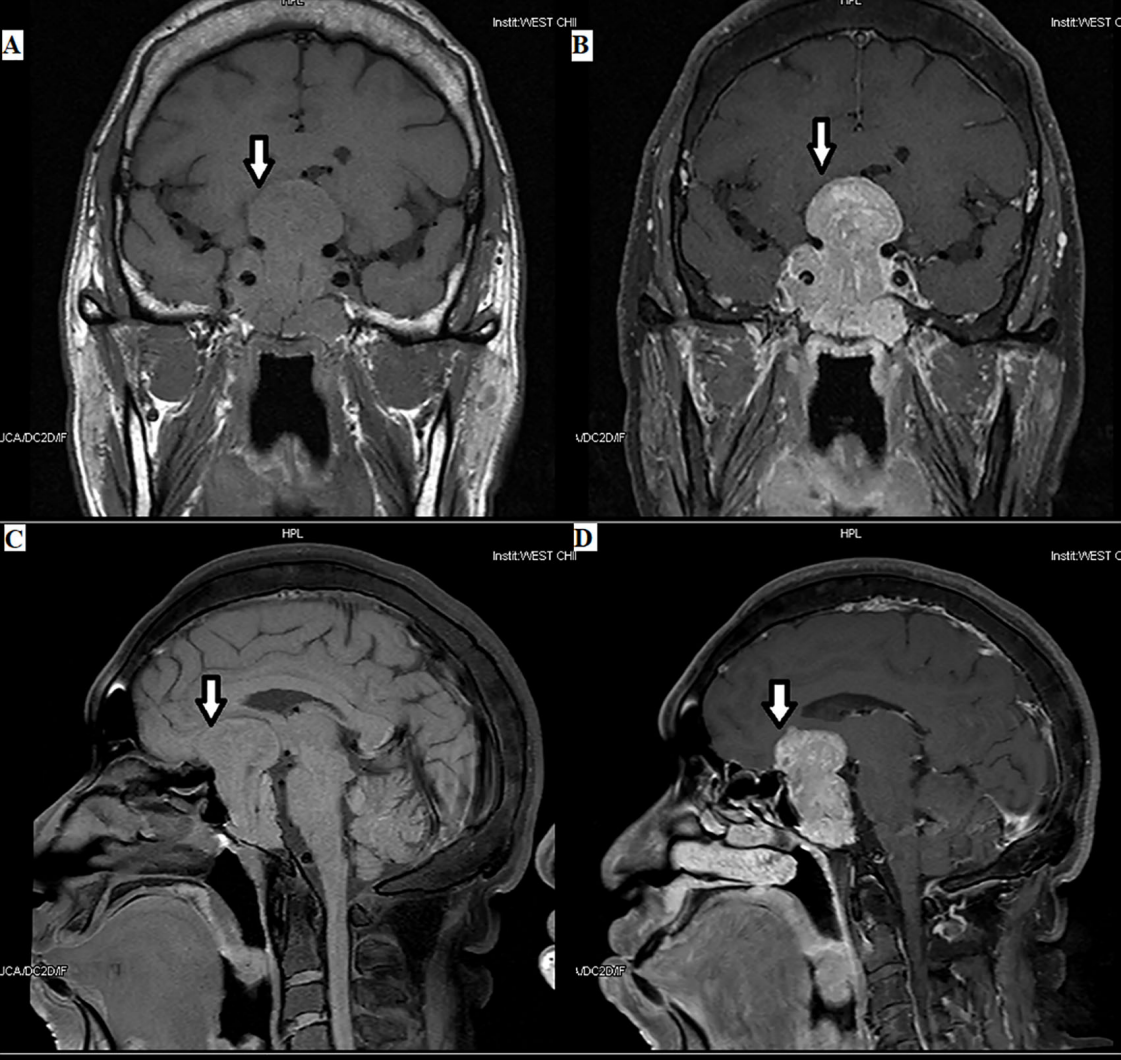
Plurihormonal TSH PitNET [growth hormone (GH) and TSH]: the tumor can be seen in the optic chiasm, hypothalamus, and saddle area, with a size of 3.8×5.6×3.5 cm, as indicated by the arrows.

Imaging examinations can not only confirm the presence of pituitary TSH PitNET but also help to understand the relationship between the tumor and the surrounding tissue (**Figures 1**, **2**). Tumors in the size of over 10 mm account for approximately 80%–85% of TSH PitNETs. In addition, if MRI or CT does not reveal any tumor in the sella turcica and laboratory tests indicate a TSH PitNET, attention should be paid to the screening of ectopic tumors despite their rarity. Since 1996, when Cooper et al. (34) first reported ectopic TSH PitNETs, all ectopic TSH PitNETs reported were located in the extracranial nasopharyngeal region. In 2016, Wang et al. (35) reported the first ectopic TSH PitNET located on the intracranial sella. Ectopic TSH PitNETs located on sphenoid sinus and orbit have been respectively first reported in 2020 (36).

Tumors with a diameter of 3~10 mm can be found by high-resolution CT or enhanced MRI in the sella turcica region. MRI is the preferred imageological examination in patients with TSH PitNETs. MRI has the characteristics of high resolution in soft tissue and noninvasive and multidirectional imaging. It can not only clearly show the shape, size, and invasion of the tumor but also accurately determine the texture of the tumor, which is conducive to making surgery plans and evaluating prognosis. With the introduction of dynamic contrast-enhanced MRI in the early 1990s, the sensitivity for diagnosing TSH PitNETs increased rapidly. For patients with suspected TSH PitNETs, a sella region MRI can be requested, and the sequences involve sagittal and coronal thin layers on T1WI and T2WI and dynamic enhanced imaging. Enhancement of high-resolution for three-dimensional imaging can improve the detection rate. The features of TSH PitNETs involve the following signs. First, TSH PitNETs are mostly rounded, ovoid, lobulated, or irregular. When combined with cystic degeneration, necrosis, hemorrhage, and calcification, it may show mixed signals. In the enhanced scanning, the substance of the tumor is intensified obviously and the portions with cystic degeneration, necrosis, hemorrhage, and calcification are not intensified. Second, it has been reported that approximately 60% of TSH PitNETs extend over the sella turcica or invade the surrounding tissue, such as the sphenoid sinus. We can find that the pituitary stalk deviates, the pituitary protrudes, and the sella turcica is locally sunken, or its bone is damaged. The pituitary gland may also dilate outward and compress the internal carotid artery.

For patients with an enormous tumor, a thin layer CT scan of the sella turcica region should be performed to observe the changes in the bone. Moreover, if patients have contraindications for an MRI, sagittal and coronal reconstruction CT in a thin-layer scan in the sella turcica region is necessary.

Thyrotroph tumors and TSH plurihormonal tumors of Pit-1 lineage have a high level of expression of SSTR, particularly SSTR 2 and 5, which makes it a good candidate for the scintigraphic test. Scintigraphy with radio-labeled octreotide can successfully localize most PitNETs. Other eikonogen can be available, including lanreotide, ^123^I, ^111^IN, ^99m^Tc, ^64^Cu-NIRF-CCPM-octreotide, ^64^Cu-DOTA-TOC, and ^64^Cu-DOTA-TATE (37). However, this technique’s specificity is low, as positive scans can occur in cases of pituitary masses of different types, either secreting or nonsecreting, and even in normal pituitary tissue. Scintigraphy tends to be irreplaceable to search for ectopic tumors when MRI or CT is unavailable. PET/CT has a superior resolution and imaging quality over scintigraphy. It can detect tumors that cannot be detected by MRI, but it has not yet been widely used due to its expensive cost and lack of study.

## Differential Diagnosis

The diagnosis of TSH PitNETs requires the identification of some physiological or pathological conditions as following. In addition, the disturbances from laboratory techniques should also be excluded.

### RTH (Thyroid Hormone Resistance Syndrome)

RTH is a disease with the autosomal dominant-inherited mutation in the β-isoform of T3 receptor, which can inhibit the interaction between T3 and its receptor and decrease the insensitivity of T3. Other gene mutations in monocarboxylate transporter 8 (MCT8) and selenocysteine insertion sequence binding protein 2 (SBP2), are related to RTH as well (38). The differentiation between the two diseases involves the following four aspects. First, in terms of clinical presentation, familial cases are more common in RTH, while TSH PitNETs occur more sporadically, which means when there is a positive familial history, RTH should be primarily suspected. However, there are about 28% of RTH patients have no family history. In a Chinese study of 61 patients with the syndrome of inappropriate secretion of TSH(SITSH), only 10% of RTH patients had a positive family history, indicating the limitation of family history for differential diagnosis (6). In addition, the thyroid goiter, the mass effect, and related presentation of increased or decreased secretion of other hormones from the anterior pituitary mentioned above are negative in RTH. Second, the difference of biochemical features show as following: 1) in patients with RTH, TRH and TSH are inappropriately increased because of the tissue resistance to T3, while in patients with TSH PitNET, TSH is normal or increased and TRH is normal, increased or decreased; 2) patients with TSH PitNET may present elevation in other pituitary hormones, SHBG, ICTP, α-SU or α-SU/TSH ratio, which cannot be seen in RTH; 3) most patients with RTH show a normal or enhanced reaction in the TRH stimulation test and the T3 inhibition test, while TSH PitNET patients show no reaction; 4) More than 90% patients with TSH PitNET show a significant decrease in TSH, FT3, and FT4 after injection of a somatostatin analogue, while the patients with RTH show a lack of response, which indicates that the somatostatin depression test and the diagnostic treatment (for at least 2 months) with somatostatin analogues can also be used for differential diagnosis (30). Third, the result of the imaging test for RTH is negative, while TSH PitNET can be detected in such tests. Fourth, TRβ gene analysis can help identify RTH, although it is more expensive and requires advanced equipment. However, 15% of RTH patients have no TRβ gene mutations (15).

### FDH (Familial Dysalbuminemic Hyperthyroxinemia)

FDH is the most common inherited cause of increased T4. It is a familial autosomal dominant disease caused by a mutation in the albumin gene, leading to the structural modification of albumin and an increased affinity between albumin and thyroxine. There are four aspects to differentiate FDH from TSH PitNETs. First, familial cases are more common in FDH, while TSH PitNETs occur more sporadically. Second, due to the increased affinity between albumin and thyroxine, the level of TT4 is elevated, but the levels of FT3, FT4, and TSH are normal. Third, FDH has no symptom of hyperthyroidism, as recombinant T4 has no biological activity. Fourth, albumin gene detection may also be used for identification.

### Graves’ Disease

There are four aspects can differentiate Graves’ disease from TSH PitNETs. First, patients with Graves’ disease usually present symptoms of hyperthyroidism, such as hyperhidrosis, heart disease, bulging eyes, and mucous edema. However, the clinical symptoms of hyperthyroidism in patients with TSH PitNETs are more slight or atypical. Second, patients with Graves’ disease show decreased TSH and positive TPOAb, TgAb, and TRAb, while patients with TSH PitNET show an increased or normal TSH and negative above antibodies. Third, the imaging tests (MRI, CT, and others) show an intracranial mass in patients with TSH PitNET, which is negative in patients with Graves’ disease. Fourth, antithyroid drugs are effective to patients with Graves’ disease but not effective to that with TSH PitNET. TSH PitNET may coexist with Graves’ disease, which makes it more difficult to diagnose.

### Chronic Lymphocytic Thyroiditis

Chronic lymphocytic thyroiditis can lead to increased TSH secretion, which also needs a differential diagnosis. First, chronic lymphocytic thyroiditis is more common in women and shows obvious family aggregation. While TSH PitNETs show no significant difference in gender and are mostly sporadic. Second, the clinical manifestations of lymphocytic thyroiditis include hypothyroidism. On the contrary, the clinical manifestations of TSH PitNETs are hyperthyroidism. In addition, it may be accompanied by other autoimmune diseases such as systemic lupus erythematosus, Sjogren’s syndrome, rheumatoid arthritis, etc. Third, as to biochemical tests, in lymphocytic thyroiditis, the thyroid function can be normal in the early stage, followed by a period of subclinical hypothyroidism. Later, it may develop into the clinical stage of hypothyroidism, with decreased T4 and increased TSH. And a small number of patients will develop Hashitoxicosis, with increased T4 and decreased or normal TSH. The symptoms are mild and can be alleviated naturally for a short time. Besides, TPOAb, TgAb, and TRAb are positive. The levels of ESR, γ-globulin IgG, and β-lipoprotein and the number of lymphocytes may be increased. In contrast, patients with TSH PitNET may present increased T4 and decreased TSH and, additionally, increased other pituitary hormones, SHBG, ICTP, α-SU, or(and) α-SU/TSH ratio. Fourth, chronic lymphocytic thyroiditis can present characteristic features in thyroid ultrasonography, including rich blood flow in lesion areas and increased lymph nodes around the thyroid (39).

In addition, about 25% to 81% of primary hypothyroidism would lead to pituitary hyperplasia, which is known as pituitary hyperplasia secondary to primary hypothyroidism (PHPH) (40). Long-term untreated primary hypothyroidism can decrease the feedback inhibition to the pituitary gland, leading to compensatory pathological hyperplasia of pituitary cells. Apart from decreased T3 and T4 and increased TSH and TRH, PHPH may also show increased PRL. As to pituitary MRI features, pituitary hyperplasia shows isointensity on TIWI, hyperintensity on T2WI, and uniform enhancement. PHPH shows no deviation in the pituitary stalk and rarely invades the cavernous sinus or skull bone. By comparison, TSH PitNETs normally show a lower signal intensity homogeneously or inhomogeneously compared with normal brain tissue, and the difference is more obvious after enhancement. With tumor progression, the pituitary stalk may be distorted or dislocated and invades the cavernous sinus or sphenoid sinus, even the optic chiasm or the internal carotid artery. The most important identification point is that after the L-thyroxine replacement therapy, the size of mass and hormone levels can be restored nearly to normal in patients with PHPH (41).

### Medical Interference

Medical interference, such as inadequate hydrocortisone replacement after surgery in Cushing’s syndrome, inappropriate levo-thyroxine replacement therapy for hypothyroidism, a medication history of estrogens or amiodarone, and others should be excluded. Therefore, a detailed medication history is necessary.

### Other Situations

Thyroxin-binding globulin (TBG) deficiency can result in decreased TT4 and thereby increased free T4. Pregnancy may also interfere with thyroid function.

## Surgical Therapy

**Figure 3:**
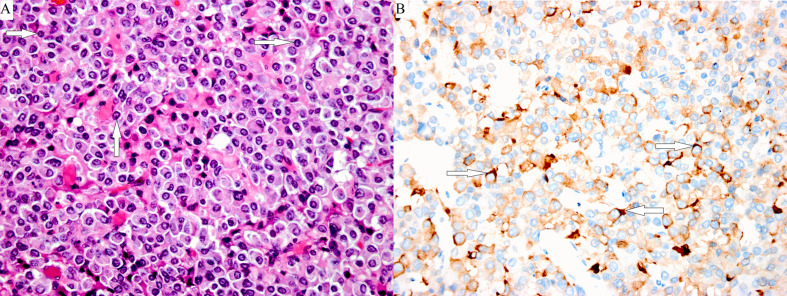
Poorly differentiated Pit-1 lineage tumor cells with chromophobic to variably eosinophilic cytoplasm and occasionally prominent nucleoli. The cell nuclei show prominent structures that correspond to spheridia, as indicated by the white arrows. (**A**: hematoxylin-eosin stain, magnification ×400). A few tumor cells were positive for TSHβ, as indicated by the white arrows (**B**: immunoperoxidase stain, magnification ×400).

Surgical therapy is the first-line treatment and is feasible for all three types of TSH PitNETs. PitNETs can be cured by surgery. Approximately 80% of patients with TSH PitNET can be improved by surgery. In addition, a pathological examination of the tumor that has been surgically removed is the gold standard for the diagnosis of TSH PitNETs (**Figure 3**). The choice of surgical approach for TSH PitNETs should aim for decompression of the optic nerve and removal of as much of the tumor as possible. The preferred treatment for TSH PitNETs is transsphenoidal surgery(TSS), for which a single-nostril transsphenoidal resection is the most common and classic method. The tumor is completely resected by TSS to preserve the normal pituitary function as much as possible. The extensive use of endoscopy enables the surgeon to observe the operative field comprehensively and avoid missing lesions, which is the first choice for the surgery. In particular, when the tumor has an obvious growth and invasion into the bottom anterior cranial region, endoscopic expansion of the transsphenoidal approach can be selected (42). Some recurrent and residual tumors also need surgery. The anatomical structure of the sella turcica region after surgery is disordered, and the scar formation in the operative field makes it difficult to distinguish the tumor from the pituitary tissue, which complicates further transsphenoidal surgery. When it is difficult to locate the position of the sella turcica bottom during surgery, intraoperative c-arm positioning, neural navigation, and intraoperative MRI can be used. Intraoperative ultrasound is valuable for the identification of the internal carotid artery for tumors invading the cavernous sinus.

Tumor invasive growth and tumor fibrosis can cause perioperative complications such as hemorrhage, cerebrospinal fluid (CSF) leakage, diabetes insipidus, and hypophysis (43). The most significant predictor of surgical success in patients is the degree of invasion into the cavernous sinuses (44).

At present, it is believed that the criteria for a complete remission of TSH PitNETs involve the disappearance of hyperthyroidism, the disappearance of neurological symptoms, the removal of the entire tumor by imaging evaluation, and normal levels of TSH, FT3, and FT4 in the blood for 3 ~ 6 months after surgery. Of course, there is no unified standard for a cure after surgery, and the factors mentioned above should be considered comprehensively.

## Radiation Therapy

Radiotherapy is generally not the preferred treatment for TSH PitNETs. With the development of drug therapy for TSH PitNETs, especially the effective use of somatostatin analogues, the use of radiotherapy has gradually been reduced. However, radiotherapy is relatively more common in poorly differentiated Pit-1 lineage tumors because it is resistant to drug therapy (13). Radiation therapy is suitable for patients who have contraindications of surgery or drugs, as well as patients with postoperative residual tumors. For patients with a postoperative complete remission, prophylactic radiotherapy is not recommended. Radiotherapy consists of traditional radiotherapy (CRT) and radiosurgery (RS) therapy (such as gamma knife, cyber knife, and others). For general radiotherapy, it is recommended to use precise radiotherapy techniques such as three-dimensional conformal radiotherapy or intensity-modulated radiotherapy. The prescribed dose at the edge of radiosurgery treatment is 12 ~ 25 Gy, and the recommended dose for a single exposure is <8 ~ 12 Gy. It is important to pay attention to protecting the optic chiasm. Some patients can be cured after radiotherapy. The major complication of radiation therapy for TSH PitNETs is hypopituitarism, which requires replacement therapy with appropriate hormones (such as levothyroxine, cortisone, or sex hormones).

## Medical Therapy

Medical therapy is effective for thyrotroph tumor and TSH plurihormonal tumors of Pit-1 lineage, while poorly differentiated Pit-1 lineage tumors are resistant. For patients with surgical contraindications or residual or recurrent tumor after surgery, medication may be considered. At present, the main drugs for TSH PitNETs are somatostatin analogues (SSAs) and dopamine receptor agonists (DA). The effect of the combination of SST and DA is uncertain. Gatto et al. (42) indicated that the combination of octreotide and cabergoline did not show any additive or synergistic effects.

Although surgery is the preferred treatment for TSH PitNETs, preoperative adjustment of thyroid function to normal levels is required. SST has been proven effective in preoperative improvement of thyroid function in most cases (45). Patients after SSA therapy can show tumor shrinkage and vision improvement. The SSTRs are expressed on TSH PitNET cells. The effect of SSAs therapy is dependent on the sensitivity of patients to Sandostatin. Studies indicate that SSA may control TSH secretion by interacting with SSTR2 and restrain cell proliferation by interacting with SSTR5. First-generation SSAs, such as octreotide and lanreotide, show a preferential affinity for SSTR2 and a moderate affinity for SSTR5. The newly available SSA, pasireotide, shows a preferential binding affinity for SSTR5 > SSTR2 > SSTR1 > SSTR3. Fang et al. (46) found that after SSAs treatment, the cell membranes were dissolved and broken, the mitochondria were swollen, the endoplasmic reticulum was elongated and liquefied, and the nucleus and organelles were dissolved. There have been no adverse consequences reported during pregnancy at present. However, due to the lack of sufficient case studies, we cannot confirm that SST is safe for pregnant women. The side effects, such as gastrointestinal discomfort, gallstones, cholecystitis, hyperglycemia, and diabetes, should be monitored (47).

Dopamine receptors (DR) are expressed on the cell membranes of thyrotrophs. Dopamine receptor agonists (bromocriptine, cabergoline) can inhibit TSH secretion in patients with TSH PitNETs, especially in patients with high prolactin. Bromocriptine therapy may control the excessive secretion of thyroid hormone in small numbers of patients. Yang et al. (48) reported a case that was unresponsive to octreotide at first, but after treatment with bromocriptine, the thyroid function returned to normal.

Antithyroid drugs (ATD) and propranolol can also be used for preoperative control of hyperthyroidism in patients with TSH PitNET, which may avoid perioperative thyroid crisis. For patients who cannot afford the high price of SST or cannot tolerate the side effects of SST and DA, ATD is an alternative. It should be noted that ATD has the following disadvantages. First, the long-term use of such drugs leads to decreased thyroid hormone levels, and negative feedback may lead to a large increase in TSH. Second, Beck-Peccoz et al. (49) reported that ATD may induce invasive growth of TSH PitNET, and thus ATD is not recommended for long-term administration.

In addition, because poorly differentiated Pit-1 lineage tumors are resistant to the drugs mentioned before, studies have raised the possibility of immune checkpoint inhibitors or nonspecific immunotherapy, which needs further research (50).

## Follow-Up After Treatment

Patients with TSH PitNET should be closely monitored after treatment (whether by surgery, radiotherapy, or medication). Before and after treatment, patients need to receive health information to understand the importance of long-term follow-up to improve their quality of life. Follow-up should be a regular assessment of the disease remission situation (including clinical manifestations, biochemical tests, imaging tests) at 3 months, 6 months, and 1 year after the operation and every year thereafter. T3 inhibition test is the most sensitive and specific test to evaluate complete tumor resection compared with other tests. TSH can also be well evaluated for complete resection. When the level of TSH remains normal one week after the operation, complete resection is more likely. However, the level of TSH is normal in 25% of TSH PitNET patients, so its sensitivity is relatively low. The specificity of indicators including FT4, FT3, SHBG, ICTP and et, al is low and temporary normality of that may only indicate transient clinical remission (15). For thyrotroph tumors and TSH plurihormonal tumors of Pit-1 lineage, recurrence is rare in the first few years after successful surgery. Biochemical remission was achieved in 66% of patients after adjuvant radiation therapy and in 76% of that after adjuvant medical treatment (25). However, poorly differentiated Pit-1 lineage tumors show a high recurrence rate and low survival rate, so follow-up should be more frequent.

## Summary

TSH PitNETs consist of at least three types of tumors, including thyrotroph tumors, poorly differentiated Pit-1 lineage tumors, and TSH plurihormonal tumors of Pit-1 lineage. It is important to understand the pathology, clinical and biochemical features, diagnosis, treatment, and prognosis of these three distinct tumors. In general, due to the development of modern imaging technology and TSH sensitivity measurements, the diagnosis of TSH PitNETs has been greatly improved. Currently, no single test is perfect. Only a combination of multiple tests can correctly identify and make a clear diagnosis. Functional test methods have not been unified and need to be further improved. At present, TSS is still the preferred treatment for most TSH PitNETs. In particular, in recent years, with the continuous development of endoscopy, an increasing number of transsphenoidal endoscopic surgeries have been carried out, which is the development trend in the future. Radiotherapy may also be an option for patients who are not in remission after surgery but cannot receive long-term medication. SSAs and DA are also widely used in the treatment of TSH PitNETs except for poorly differentiated Pit-1 lineage tumors. TSH PitNETs are clinically relatively rare and require clinicians to be on high alert. With the development of pathogenesis and multidisciplinary team (MDT) collaborative diagnosis, the combination of multiple treatments will improve the diagnosis and treatment of TSH PitNETs.

## Author Contributions

PL was the major contributor to the writing and conception of the manuscript. HT contributed to drafting the manuscript and revised it for intellectual content. LZ and LY collected the data. ZA revised the manuscript for intellectual content. All authors contributed to the manuscript and approved the submitted version.

## Funding

This study was supported by the Sichuan Province Science and Technology Project of China (Grant No. 0040205302062).

## Conflict of Interest

The authors declare that the research was conducted in the absence of any commercial or financial relationships that could be construed as a potential conflict of interest.
